# The Free Thyroxine-to-Thyroid Stimulating Hormone Ratio: A Potential Diagnostic Marker for Graves’ Disease

**DOI:** 10.5812/ijem-158565

**Published:** 2025-01-30

**Authors:** Norashidah Binti Rahmat, Tuan Salwani Tuan Ismail, Mohd Zakwan bin Md Muslim, Wan Mohd Saifuhisam bin Wan Zain, Adlin Zafrulan bin Zakaria, Mohd Yusran bin Yusoff

**Affiliations:** 1 Chemical Pathology Unit, Department of Pathology, Hospital Raja Perempuan Zainab II, Kota Bharu, Kelantan, Malaysia; 2 Endocrinology Unit, Chemical Pathology Department, School of Medical Sciences, Universiti Sains Malaysia, 16150 Kota Bharu, Malaysia; 3 Hospital Pakar Sultanah Fatimah, Muar, Johor, Malaysia; 4 Department of Chemical Pathology, School of Medical Sciences, Universiti Sains Malaysia Health Campus, Kelantan, Malaysia; 5 Universiti Sains Malaysia Specialist Hospital, Kelantan, Malaysia; 6 Department of Pathology, Hospital Raja Perempuan Zainab II, Kota Bharu, Kelantan, Malaysia; 7 CRC Kelantan, Institute for Clinical Research, National Institute of Health, Ministry of Health Malaysia, Putrajaya, Malaysia

**Keywords:** Graves' Disease, FT4/TSH Ratio, Diagnostic Accuracy

## Abstract

**Background:**

Graves' disease (GD) is a leading cause of hyperthyroidism, characterized by excessive thyroid hormone production. Although the thyroid stimulating hormone receptor autoantibodies (TRAb) test is specific for GD, its limited accessibility often delays diagnosis and treatment, leading to potential complications. Thus, exploring alternative diagnostic markers, such as thyroid hormone ratios, may offer a feasible solution.

**Objectives:**

This study aims to assess the diagnostic accuracy of the free thyroxine-to-thyroid stimulating hormone (FT4/TSH) ratio in distinguishing GD from other non-Graves’ disease (NGD) hyperthyroidism.

**Methods:**

A retrospective study was conducted at Hospital Raja Perempuan Zainab II in Kelantan, Malaysia, from 2021 to 2023. A total of 351 hyperthyroid patients who underwent initial TRAb testing during this period were included. These patients were categorized into two groups: Graves' disease and NGD hyperthyroidism, based on definitive diagnoses made by endocrinologists, as documented in the electronic medical records. Data on patients' TSH, FT4, and FT4/TSH ratios and TRAb results were retrieved from the laboratory information system (LIS) for analysis. The diagnostic accuracy of these parameters was assessed using receiver operating characteristic (ROC) curve analysis to determine optimal cut-off values, sensitivity, specificity, positive predictive values (PPVs), and negative predictive values (NPVs).

**Results:**

Patients with GD had significantly higher FT4 and FT4/TSH ratios and lower TSH levels than NGD hyperthyroid patients (P < 0.001). Receiver operating characteristic analysis identified an FT4/TSH ratio cut-off of 13948.98 pmol/mIU, yielding a specificity of 99.4%, PPV of 92.31%, and an area under the curve (AUC) of 0.740.

**Conclusions:**

The FT4/TSH ratio shows promise as an accessible diagnostic marker for GD, particularly where TRAb testing is limited. Its high specificity and PPV could facilitate timely diagnosis, improving patient management and outcomes. Further studies are needed to validate this approach in larger populations.

## 1. Background

Graves' disease (GD), an autoimmune thyroid disorder, is the leading cause of hyperthyroidism and is characterized by excessive production of thyroid hormones (TH), primarily thyroxine (T4) and triiodothyronine (T3). The resulting thyrotoxicosis manifests with symptoms such as palpitations and heat intolerance. Diagnosis typically relies on thyroid stimulating hormone receptor autoantibodies (TRAb), a specific biomarker for GD (1, 2). However, TRAb analysis is performed in centralized laboratories in many health facilities and is selectively ordered, usually by specialists or endocrinologists. This often leads to delays in diagnosis and treatment initiation, potentially worsening complications such as thyroid storm and osteoporosis (3).

The European Thyroid Association (ETA) recommends a combination of clinical evaluation—symptoms, stigmata (such as exophthalmos)—and biochemical markers (4). While TRAb testing is critical for confirming GD and distinguishing it from thyroiditis (2), limitations such as cost, accessibility, and occasional false-negative results, particularly in atypical or mild cases, are well documented (5).

At many referral centers, including ours, TRAb measurement is routinely performed using advanced immunoassay-based techniques, specifically electrochemiluminescence immunoassay (ECLIA) (6). While ECLIA has significantly improved TRAb detection, diagnostic challenges persist due to lower specificity at commonly used cut-offs (7). The manufacturer-recommended cut-off of 1.75 IU/L for TRAb positivity, used to differentiate GD from non-GD (NGD) hyperthyroidism, achieves a specificity of only 62.96% (7), increasing the risk of false positives. Research by John et al. demonstrated that raising the cut-off to 3.37 IU/L significantly improves specificity to 90.12% (7), enhancing diagnostic accuracy while maintaining clinical relevance.

Alternative diagnostic methods such as radioactive iodine uptake (RAIU) or thyroid scintigraphy are invasive, costly, and unsuitable for specific populations, including pregnant women (1). These limitations highlight the need for faster, non-invasive, and cost-effective diagnostic tools.

Emerging evidence suggests that the free T3-to-thyroid stimulating hormone (FT3/TSH) ratio (8) and the FT3-to-free thyroxine (FT3/FT4) ratio (9-12) may offer viable alternatives for diagnosing GD. Additionally, research by Alidrisi et al. highlighted the T3/FT4 ratio as a valuable tool for distinguishing GD from subacute thyroiditis (13). By increasing the T3/FT4 ratio cut-off to 100 or higher, specificity and positive predictive value (PPV) reached up to 100% (13). However, findings remain inconsistent, and these ratios are not widely adopted in clinical practice due to limited availability of FT3 testing.

The FT4/TSH ratio has shown promise as a practical diagnostic tool for GD, with a reported diagnostic accuracy of up to 79.4% in distinguishing GD from subacute thyroiditis (14). Since TSH and FT4 are routinely measured in our clinical practice, the FT4/TSH ratio could offer a feasible alternative to TRAb testing, particularly in settings with limited access to TRAb.

## 2. Objectives

This study aims to assess the diagnostic accuracy of FT4, TSH, and the FT4/TSH ratio as biomarkers for diagnosing GD and to determine their effectiveness in differentiating GD from NGD hyperthyroidism. By identifying reliable and precise diagnostic markers, this study seeks to facilitate early and accurate detection of GD, thereby improving patient management and clinical outcomes.

## 3. Methods

### 3.1. Study Design

A retrospective study was conducted at Hospital Raja Perempuan Zainab II, Kota Bharu, Kelantan, Malaysia, from 1st January 2021 to 31st December 2023. Hyperthyroid patients tested for TRAb were identified using the laboratory information system (LIS), from which their TRAb, FT4, FT3, and TSH results were retrieved. Definitive diagnoses and demographic information were obtained from electronic medical records to ensure comprehensive data collection for analysis.

This study strictly adhered to the principles of the Declaration of Helsinki and received ethical approval from the Medical Research Ethics Committee (approval No: 24-02097-ZUJ (1)) and the Human Research Ethics Committee USM (code: USM/JEPeM/KK/24070587). Only the initial TRAb test for each patient was included in the analysis. This approach ensured that diagnostic accuracy was based solely on the first test result, thereby eliminating bias from prior interventions.

### 3.2. Participants

**Figure 1.**
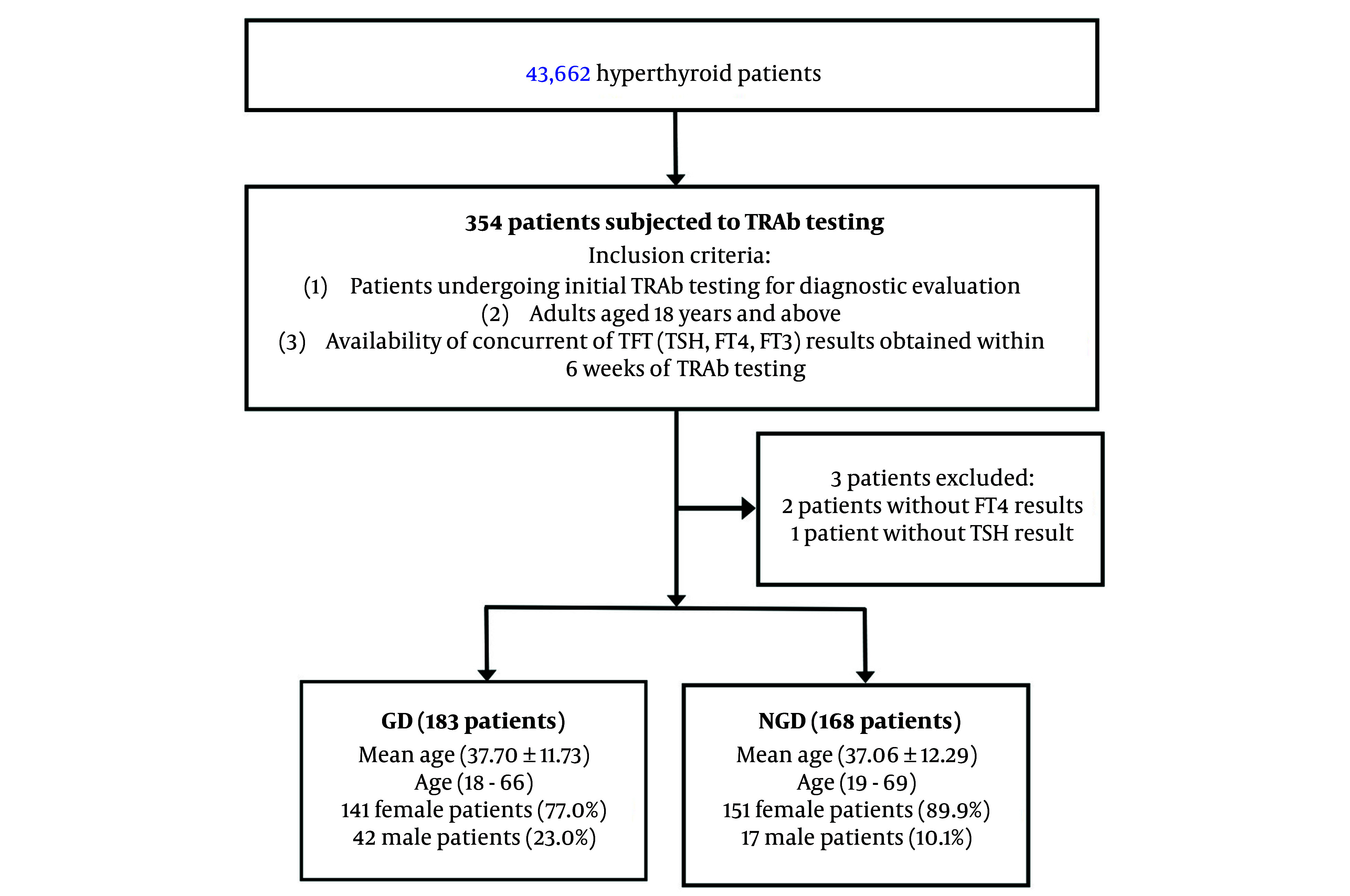
Flowchart illustrating participants selection

We examined all hyperthyroid patients during the study period. We selected adult patients (≥ 18 years) from this cohort who underwent TRAb testing for the first time. We assessed their TRAb results and the corresponding thyroid function test (TFT), including FT4, FT3, and TSH levels. Only patients with concurrent TFT results within six weeks of TRAb testing were included. Exclusion criteria were as follows: (1) Patients without concurrent TFT results obtained within six weeks of TRAb testing; (2) patients with a history of anti-thyroid medications use or at the time of sampling, thyroidectomy or radioiodine therapy, as these factors could interfere with TFT results and diagnostic accuracy; (3) patients with incomplete or insufficient data documentation for definitive diagnostic verification; (4) pregnant patients due to potential physiological alterations in thyroid function; (5) patients with palpable thyroid nodules as these may indicate additional thyroid pathology that could confound results; (6) patients with known conditions or laboratory evidence of serum protein abnormalities such as hypoalbuminaemia, that could interfere with FT4 measurement; (7) Inpatients, as severe illnesses such as sepsis are associated with non-thyroidal illness syndrome, can lead to reduced FT4 levels due to altered TH metabolism and binding protein concentrations; and (8) Patient with conditions known to cause false low FT4 levels, such as nephrotic syndrome and chronic liver disease (which reduce FT4 binding) or patients taking medications like phenytoin and rifampin (which increase FT4 clearance). A strict data validation protocol was applied to ensure all participants met the inclusion criteria. These steps were implemented to enhance the reliability and accuracy of the study findings. The overall study workflow is summarised in Figure 1. 

### 3.3. Laboratory Tests

Thyroid hormone levels—including TSH, FT4, and FT3—were measured using the DxI 800 chemiluminescence immunoassay system (Beckman Coulter) with the following reference ranges: Serum TSH (0.40 - 4.00 mIU/L), serum FT4 (7.86 - 14.41 pmol/L), and serum FT3 (3.8 - 6.0 pmol/L). Serum TRAb levels were assessed using the Cobas 801 electrochemiluminescence analyzer (Roche Diagnostics), with a reference range of < 1.75 IU/L. The lower detection limit for serum TSH was 0.005 mIU/L, while the maximum measurable concentrations for serum FT4 and FT3 were 77.2 pmol/L and 56.0 pmol/L, respectively.

The analytical performance of the TFTs used throughout the study was assessed based on their coefficient of variation (CV), a key indicator of assay precision. The intra-assay CV for TSH ranged from 4.14% to 5.47%, and the inter-assay CV ranged from 4.23% to 7.12%. For FT4, the intra-assay CV ranged from 3.57% to 7.42%, and the inter-assay CV ranged from 4.61% to 6.84%. For FT3, the intra-assay CV ranged from 6.85% to 9.71%, and the inter-assay CV ranged from 5.49% to 8.96%.

The intra-assay CV for TRAb ranged from 4.42% to 6.45%, while the inter-assay CV ranged from 4.97% to 7.31%. These CV values fall within the acceptable limits defined by analytical performance specifications (APS) for clinical diagnostic laboratories, thereby ensuring the reliability and reproducibility of the results.

### 3.4. Diagnosis of GD

The diagnosis of GD was determined by an endocrinologist based on a positive TRAb test and clinical evaluation. The diagnostic criteria followed the 2018 ETA guideline for GD, which includes:

(1) Clinical symptoms and signs of hypermetabolism secondary to thyrotoxicosis,

(2) Diffuse thyroid enlargement confirmed by palpation or ultrasound,

(3) Thyroid function test results showing elevated FT3 and/or FT4 levels with a suppressed TSH level, and

(4) Stigmata of GD such as exophthalmos, other ocular manifestations, and pretibial myxoedema (4).

A positive TRAb test is considered a key diagnostic marker for GD (4). Supplemental diagnostic indicators included RAIU testing or increased uptake observed in ^99^mTcO4 thyroid scintigraphy to establish etiological evidence and aid in the differential diagnosis from other causes of thyrotoxicosis (4).

### 3.5. Statistical Analysis

Data were analyzed using the statistical package for the social sciences (SPSS) version 28.0 (IBM SPSS Statistics). Categorical variables were summarized as frequencies (No.) and percentages (%). Continuous variables were presented as means ± standard deviations for normally distributed data, or as medians with interquartile ranges (IQR) for non-normally distributed data.

Receiver operating characteristic (ROC) curves were generated to determine the optimal cut-off values, sensitivity, specificity, PPV, negative predictive value (NPV), and overall diagnostic accuracy of FT4, TSH, and the FT4/TSH ratio in diagnosing GD. A P-value < 0.05 was considered statistically significant. Spearman’s correlation analysis was used to assess relationships between variables.

## 4. Results

### 4.1. Demographic Data

Table 1 presents the demographic characteristics of the study population. A total of 351 untreated patients who underwent initial TRAb testing were included, with ages ranging from 18 to 69 years. The majority of the participants were women (83.5%). Among them, 183 patients tested TRAb-positive and were diagnosed with GD, while 168 TRAb-negative patients comprised the NGD hyperthyroid group.

**Table 1.** Socio-Demographic Characteristics of Study Participants ^a^

Variables	GD (n = 183)	NGD Hyperthyroidism (n = 168)
**Age**	37.70 ± 11.73	37.06 ± 12.29
**Gender**		
Female	141 (77.0)	151 (89.9)
Male	42 (23.0)	17 (10.1)
**Race**		
Malay	174 (95.1)	158 (94.0)
Chinese	8 (4.4)	4 (2.4)
Indian	0 (0.0)	4 (2.4)
Siamese	1 (0.5)	2 (1.2)
**Status**		
Outpatient	118 (64.5)	94 (56.0)
Inpatient	65 (35.5)	74 (44.0)
**Patient’s residence**		
City (urban)	117 (63.9)	113 (67.3)
District (rural)	66 (36.1)	55 (32.7)

Abbreviations: GD, Graves’ disease; NGD, non-GD.

^a^ Values are expressed as mean ± SD or No. (%).

### 4.2. TSH, FT4, FT3 Levels and FT3/FT4, FT4/TSH and FT3/TSH Ratios

All patients with GD exhibited significantly higher FT4 levels and FT4/TSH ratios, as well as significantly lower TSH levels (P < 0.001 for all), as shown in Table 2. However, FT3 levels, FT3/FT4 ratios, and FT3/TSH ratios did not demonstrate significant differences between the GD and NGD hyperthyroid groups.

**Table 2.** Comparison of the Baseline Thyroid Function Data Between Graves’ Disease and Non-Graves’ Disease Hyperthyroidism ^a^

Variables	GD	NGD	Z Statistic	P-Value
**TSH (mIU/L)**	0.01 (0.01)	0.04 (1.11)	-7.62	< 0.001 ^b^
**FT4 (pmol/L)**	17.10 (30.80)	12.80 (7.15)	-4.68	< 0.001 ^b^
**FT3 (pmol/L)**	5.30 (1.25)	5.30 (1.17)	-1.02	0.309
**FT3/FT4 (pmol/pmol)**	0.46 (0.17)	0.45 (0.13)	-0.43	0.669
**FT4/TSH (pmol/mIU)**	3428.57 (7061.84)	448.75 (2821.69)	-7.77	< 0.001 ^b^
**FT3/TSH (pmol/mIU)**	887.76 (829.95)	505.00 (1035.26)	-1.45	0.146
**TRAb (IU/L)**	7.02 (9.80)	0.79 (0.00)	-10.04	< 0.001 ^b^

Abbreviations: GD, Graves’ disease; NGD, non-GD; IQR, interquartile ranges; TSH, thyroid stimulating hormone; FT4, free thyroxine; FT3, free triiodothyronine; TRAb, thyroid stimulating hormone receptor autoantibodies.

^a^ Values are expressed as median (IQR)

^b^ Statistically significant (P < 0.05).

The median TSH level in the GD group was 0.01 mIU/L, while the lower detection limit of the serum TSH assay used in this study was 0.005 mIU/L. This highly sensitive assay accurately detects and differentiates TSH values below 0.01 mIU/L, ensuring the precision and reliability of FT4/TSH ratio calculations. The assay's sensitivity minimizes variability due to detectability constraints, underscoring the robustness of our methodology and supporting the validity of the FT4/TSH ratio in distinguishing GD from NGD hyperthyroid cases.

### 4.3. Diagnostic Value of TSH, FT3, FT4 Levels, and FT3/FT4, FT4/TSH and FT3/TSH Ratios in Diagnosing GD

**Figure 2.**
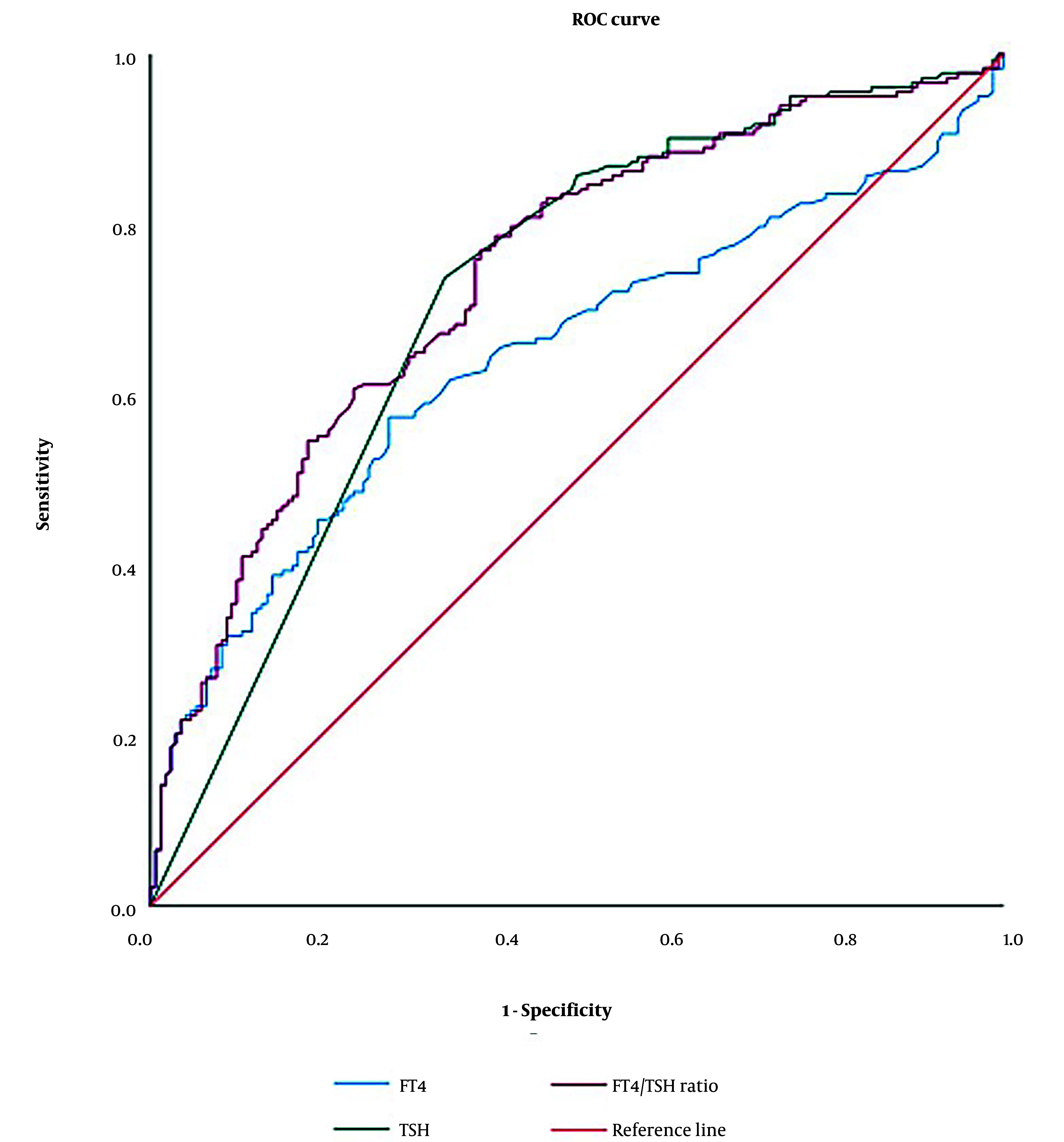
Receiver operating characteristic (ROC) curve analysis for free thyroxine (FT4), thyroid-stimulating hormone (TSH) and FT4/TSH in diagnosing Graves’ disease

We performed a ROC curve analysis to determine the diagnostic cut-off values for FT4, TSH, and the FT4/TSH ratio in distinguishing GD from NGD hyperthyroidism (Figure 2). The ROC curve is a graphical representation of a diagnostic test's performance, plotting sensitivity (true positive rate) against 1-specificity (false positive rate) across various threshold settings to identify the optimal cut-off value.

For FT4, the optimal cut-off value was 68.35 pmol/L, yielding a sensitivity of 6.6% and a specificity of 99.4%, with an area under the curve (AUC) of 0.645 (95% confidence interval [CI]: 0.587 - 0.703). For TSH, the optimal cut-off value was 0.007 mIU/L, providing a sensitivity of 73.8%, specificity of 65.5%, and an AUC of 0.715 (95% CI: 0.660 - 0.770). The FT4/TSH ratio demonstrated the highest diagnostic value, with an optimal cut-off of 13948.98 pmol/mIU, yielding a sensitivity of 6.6%, specificity of 99.4%, and an AUC of 0.740 (95% CI: 0.688 - 0.792).

Positive predictive values and NPVs were calculated based on these optimal cut-offs (Table 3). Both the FT4/TSH ratio and FT4 alone demonstrated superior specificity and PPV compared to TSH alone. Notably, the FT4/TSH ratio exhibited the highest AUC, underscoring its potential as a more reliable indicator for diagnosing GD.

**Table 3.** Results of Each Parameter in the Diagnosis of Graves’ Disease

Parameters	AUC	95% CI	Cut-off	SS (%)	SP (%)	PPV (%)	NPV (%)	DA (%)
**FT4 (pmol/L)**	0.645	0.587 - 0.703	> 68.35	6.6	99.4	92.3	49.4	51
**TSH (mIU/L)**	0.715	0.660 - 0.770	< 0.007	73.8	65.5	70.0	69.6	70
**FT4/TSH (pmol/mIU)**	0.740	0.688 - 0.792	>13948.98	6.6	99.4	92.3	49.4	51

Abbreviations: AUC, area under curve; CI, confidence interval; SS, sensitivity; SP, specificity; PPV, positive predictive value; NPV, negative predictive value; DA, diagnostic accuracy; FT4, free thyroxine; TSH, thyroid stimulating hormone.

**Figure 3.**
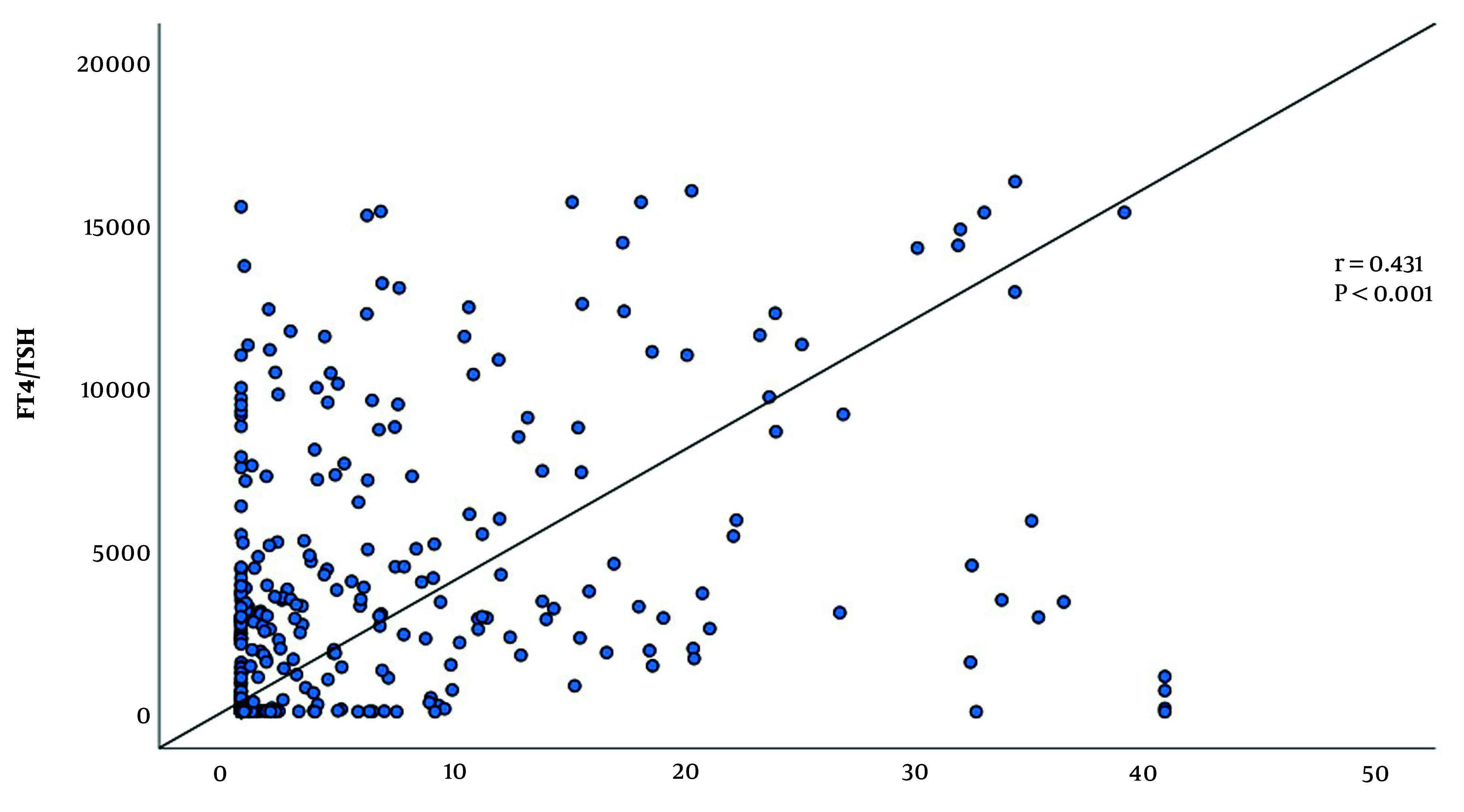
Correlation between free thyroxine (FT4)/thyroid stimulating hormone (TSH) and thyroid stimulating hormone receptor autoantibodies (TRAb) titers

We then analyzed the FT4/TSH ratio in GD patients stratified by TRAb titer levels. As illustrated in Figure 3, the FT4/TSH ratio increased in parallel with rising TRAb titers among GD patients. Correlation analysis demonstrated a statistically significant positive correlation between the FT4/TSH ratio and TRAb levels (r = 0.431, P < 0.001), further supporting the association between TRAb activity and TH dysregulation (Figure 3). 

## 5. Discussion

Our findings highlight the diagnostic value of the FT4/TSH ratio in GD, demonstrating its high specificity (99.4%) and PPV of 92.3%, with an AUC of 0.740. These results position the FT4/TSH ratio as a valuable, non-invasive alternative diagnostic marker for GD—particularly in resource-limited settings where access to TRAb testing is constrained. Although the FT4/TSH ratio showed lower sensitivity, its diagnostic utility can be significantly enhanced when combined with clinical symptoms, imaging studies, and other supportive diagnostic indicators.

While both FT4 and the FT4/TSH ratio exhibited comparable specificity, the notably higher AUC observed for the FT4/TSH ratio underscores its superior diagnostic potential. Furthermore, the strong positive correlation between FT4/TSH ratio and TRAb levels found in our study supports its role as a reliable surrogate biomarker in the diagnosis of GD.

Interestingly, our analysis revealed that FT3, FT3/FT4, and FT3/TSH ratios did not reach statistical significance (P > 0.05), indicating that these markers may not be ideal for diagnosing GD. This may be attributed to the smaller sample size and the limited routine use of FT3 testing. Additionally, the higher cost of FT3 assays, as compared to TSH and FT4, restricts their application to selective, reflective testing in specific clinical scenarios. This finding further emphasizes the practicality and accessibility of FT4 and TSH measurements, which are widely available and routinely requested in thyroid function assessments across Malaysia.

In the current healthcare landscape, timely and accurate diagnosis of GD is essential for improving patient outcomes. In Malaysian public healthcare settings, TRAb testing is centralized and processed through a single reference center to optimize costs. However, this approach results in significant delays—often exceeding three months—before results are available. These logistical challenges reinforce the urgent need for reliable, rapid, and cost-effective diagnostic alternatives that can be readily adopted in decentralized healthcare facilities.

Previous studies have explored the diagnostic potential of ratios such as FT3/FT4 (9-13) and FT3/TSH (8) in differentiating GD from other thyroid disorders. A recent study by Khan et al. (conducted from November 2023 to May 2024) reported that the FT3/FT4 ratio achieved a sensitivity of 87%, specificity of 85%, and an AUC of 0.92 in distinguishing GD from subacute thyroiditis (15). Similarly, another study found an AUC of 0.936 for the FT3/FT4 ratio, with sensitivity and specificity values of 89.7% and 91.8%, respectively (16). Despite these promising results, the studies were limited by small sample sizes and single-center designs, potentially restricting the generalizability of their findings.

Moreover, there is variability in the diagnostic confirmation methods employed across studies. For example, a study by Ibrahim and Hamam (16) used confirmatory imaging techniques such as Technetium-99m thyroid scans, while others relied on TRAb testing. This lack of standardization underscores the need for consistent diagnostic criteria in future research to better validate the diagnostic performance of hormone ratios in GD.

In contrast, Shigemasa et al. demonstrated that the total T3/T4 ratio effectively distinguishes GD from painless thyroiditis (PT) (17). However, the FT3/FT4 ratio showed significant overlap between the two groups (17), making it a less reliable diagnostic marker. This discrepancy arises because the T3/T4 ratio reflects total hormone levels (both bound and unbound), which are influenced by variations in thyroxine-binding globulin (TBG) and prealbumin (TBPA). In contrast, the FT3/FT4 ratio focuses exclusively on unbound hormones, which are more directly affected by changes in binding capacity.

Our findings align with those of Zhang et al. in China, who identified the FT4/TSH ratio as a valuable diagnostic marker for GD. Their study reported an AUC of 0.846 (14), with high specificity (90.16%), sensitivity (71.52%), a PPV of 90.77%, and an overall diagnostic accuracy of 79.44% at a cut-off value of > 5731.286 pmol/mIU. Similarly, an earlier five-year study (2015 - 2019) demonstrated that the FT4/TSH ratio was statistically significant (P < 0.0001) in differentiating GD from autoimmune thyroiditis (8). That study reported an AUC of 0.83 (95% CI: 0.81 - 0.86, P < 0.0001), with an optimal cut-off of 5.99 pmol/mIU, achieving 76% sensitivity and 75% specificity.

A key distinction between our study and these prior investigations lies in the higher specificity and PPV observed in our findings. High specificity is essential for minimizing false-positive results, which helps prevent misdiagnosis and unnecessary treatment with anti-thyroid medications. This not only alleviates psychological stress for patients and their families but also enhances the accuracy of clinical decision-making. Furthermore, the high PPV indicates that most positive test results in our study are true positives, offering clinicians greater diagnostic confidence while reducing the need for additional confirmatory testing. As a result, healthcare resources are conserved, and timely, appropriate management of GD is facilitated.

While previous studies often relied on RAIU or ^99^mTcO4 thyroid scintigraphy to confirm GD, these methods are resource-intensive and not universally accessible. Additionally, those studies were conducted exclusively in populations of Chinese ethnicity, highlighting the importance of population-specific research. Genetic and environmental factors can influence optimal diagnostic thresholds, reinforcing the relevance of studies such as ours that focus on Southeast Asian populations. Our study also benefits from a larger sample size, which improves statistical power, reduces bias, and enhances the reliability of the findings.

In our cohort, GD cases were characterized by significantly elevated FT4 levels and TSH values near the assay's lower detection limit, resulting in markedly elevated FT4/TSH ratios. This pattern reinforces the utility of the FT4/TSH ratio as a clear marker of GD, especially in distinguishing it from NGD hyperthyroidism—an essential clinical consideration. Given the prolonged delays in TRAb test availability in Malaysia, the FT4/TSH ratio serves as a valuable and timely diagnostic alternative.

Minimizing dependence on TRAb testing allows clinicians to reduce costs, expedite diagnosis, and improve patient care by avoiding the risks associated with delayed intervention. While TRAb assays are highly sensitive, their lower specificity at commonly used cut-off values may lead to false-positive results (7). Although increasing the TRAb cut-off improves specificity, the FT4/TSH ratio provides a simpler, more efficient, and cost-effective alternative. It maintains diagnostic reliability while reducing the need for additional confirmatory testing.

Our study employed a third-generation chemiluminescence immunoassay for TSH and FT4 measurements, minimizing interference from protein-binding abnormalities such as hypoalbuminemia or low TBG. This approach reduced the risk of falsely low FT4 results. The assays demonstrated high precision, with consistently low coefficients of variation (CVs), thereby ensuring reliable and reproducible data.

The high-sensitivity TSH assay, with a detection limit of 0.005 mIU/L, accurately identified the suppressed TSH levels characteristic of thyrotoxicosis, effectively capturing the strong inhibitory feedback exerted by elevated FT4 on TSH in GD. Diagnostic accuracy was further enhanced by correlating clinical findings with TRAb positivity, minimizing the risk of falsely low TSH values. To maintain measurement precision, patients with known protein-binding abnormalities were excluded from the study.

The FT4/TSH ratio cut-off was derived from robust FT4 and TSH measurements. Notably, the median TSH value in the study cohort was 0.01 mIU/L, well above the assay’s detection limit, ensuring precise ratio calculations and reinforcing diagnostic reliability.

A key strength of our study is the use of a well-defined patient cohort and standardized FT4 and TSH measurements, which significantly enhance the reliability and reproducibility of the findings. The application of stringent inclusion and exclusion criteria helped to minimize potential confounding factors, ensuring that the observed association between the FT4/TSH ratio and GD was clinically meaningful.

However, several limitations must be acknowledged. The retrospective design may introduce bias, as data were collected from existing records rather than through a prospective approach. Additionally, the small sample size for FT3 measurements limited the statistical power to draw definitive conclusions regarding the diagnostic utility of FT3/FT4 and FT3/TSH ratios. The study population, drawn exclusively from the East Coast of Malaysia, may not fully reflect the ethnic and genetic diversity of the broader Malaysian population, highlighting the need for larger, multi-centre studies to enhance the generalizability of the results.

While the FT4/TSH ratio proved highly effective for diagnosing GD, its reliability may diminish in cases of subclinical hyperthyroidism or when TSH suppression is mild. In such instances, TRAb levels may be normal or only slightly elevated. Moreover, the FT4/TSH ratio alone cannot distinguish GD from other causes of NGD hyperthyroidism, such as toxic multinodular goitre or thyroiditis, which share overlapping clinical features but necessitate different management approaches.

Free thyroxine remains a highly sensitive marker of TH feedback, especially when TSH levels are markedly suppressed (14). However, FT4 readings can be affected by conditions such as hypoalbuminemia or altered TBG levels. One limitation of our study is the absence of total T4 measurements, which would have provided a means to validate FT4 accuracy. Correlating FT4 with total T4 is recommended, as discrepancies between the two can indicate underlying protein-binding issues.

Future studies should incorporate total T4 assessments to better evaluate the accuracy of FT4 assays. Additionally, prospective, multi-centre research involving diverse ethnicities, age groups, and varying disease severities is essential to validate the FT4/TSH ratio as a diagnostic tool for GD. Expanding the diagnostic framework to include non-invasive modalities such as thyroid ultrasonography or scintigraphy, in conjunction with the FT4/TSH ratio, may further refine diagnostic algorithms. Future investigations should also prioritize differentiating GD from conditions with overlapping presentations, such as subacute thyroiditis, which have distinct etiologies and therapeutic implications (14).

### 5.1. Conclusions

This study highlights the FT4/TSH ratio as a promising diagnostic tool for the early identification of GD, particularly in healthcare settings where access to TRAb testing is limited or delayed. By streamlining the diagnostic process, the FT4/TSH ratio offers the potential to facilitate faster, more efficient clinical decision-making, ultimately contributing to improved management and patient outcomes in GD.

## Data Availability

The dataset presented in the study is available on request from the corresponding author during submission or after publication.
